# Exonized *Alu* repeats in the 3’UTR of a *CYP20A1_Alu*-LT transcript act as a miRNA sponge

**DOI:** 10.1186/s13104-023-06289-z

**Published:** 2023-03-09

**Authors:** Khushboo Singhal, Sonam Dhamija, Mitali Mukerji

**Affiliations:** 1 CSIR-Institute of Genomics and Integrative Biology, Mathura Road, 110025 New Delhi, India; 2 Academy of Scientific and Innovative Research (AcSIR), 201002 Ghaziabad, Uttar Pradesh India; 3 Department of Bioscience and Bioengineering, Indian Institute of Technology, 342037 Jodhpur, Rajasthan India

**Keywords:** *Alu* repeats, *Alu* exonization, *GAP43*, *CYP20A1*, miRNA sponge

## Abstract

**Objective:**

*Alu* repeats have gained huge importance in the creation and modification of regulatory networks. We previously reported a unique isoform of human *CYP20A1* i.e. *CYP20A1_Alu*-LT with 23 *Alu* repeats exonized in its 9 kb long 3’UTR with 4742 potential binding sites for 994 miRNAs. The role of this transcript was hypothesized as a potential miRNA sponge in primary neurons as its expression correlated with that of 380 genes having shared miRNA sites and enriched in neuro-coagulopathy. This study provides experimental evidence for the miRNA sponge activity of *CYP20A1_Alu*-LT in neuronal cell lines.

**Results:**

We studied the *Alu*-rich fragment of the *CYP20A1_Alu*-LT extended 3’UTR with > 10 binding sites for miR-619-5p and miR-3677-3p. Enrichment of the *Alu-*rich fragment with Ago2 confirmed miRNA association of this transcript. Cloning the fragment downstream of a reporter gene led to a 90% decrease in luciferase activity. Overexpression and knockdown studies revealed a positive correlation between the expression of CYP20A1_Alu-LT and miR-619-5p / miR-3677-3p target genes. *GAP43*, one of the key modulators of nerve regeneration, was significantly altered by the expression of *CYP20A1_Alu*-LT. This study, for the first time, provides evidence for a unique regulatory function of exonized *Alu* repeats as miRNA sponges.

**Supplementary Information:**

The online version contains supplementary material available at 10.1186/s13104-023-06289-z.

## Introduction

The repertoire of primate-specific *Alu* repeats in gene regulation is ever-growing [1–4]. Exonized Alus are present in ~ 14% of human transcripts, majorly in the 3’UTRs [5–9]. They have been shown to play an important role in neuron regeneration and neuronal pathways thus also affecting the evolution of these pathways in a lineage-specific manner [10], [11]. miRNA has been shown to selectively target transcripts harboring exonized *Alus* under stress conditions [12]. Interestingly, free *Alu* RNAs have been shown to sequester miRNA-566 leading to epithelial-to-mesenchymal transition (EMT) in cancer cell lines [13].

In a previous study, we have shown that the transcript isoform of the *CYP20A1* gene i.e. *CYP20A1_Alu*-LT has 23 exonized *Alus* in its 9 kb long 3’UTR with 4742 predicted miRNA binding sites (MREs) for 994 miRNAs. ~95% of the miRNA binding sites (i.e. 4500 MREs) are present within exonized *Alus* [14]. Since there are more number of MREs than miRNAs, it is obvious that a single miRNA can have multiple MREs. In these 994 different miRNAs, when we applied a cut of > = 10 MREs for a single miRNA present on the UTR, we found that there are 140 such miRNAs with ~ 3000 sites on the entire 3’UTR. This isoform was expressed in multiple cell lines like SK-N-SH, MCF-7, HEK293, A549, and HeLa and localized to the cytoplasm. As neuronal cells prefer transcripts with long 3’UTRs [15], the characterization of the transcript was carried out in primary neurons. *CYP20A1_Alu*-LT was upregulated in the presence of HIV-TAT (an apoptotic trigger) and downregulated upon heat shock. Among the differentially expressed genes in the two conditions, a set of 380 genes enriched in neuro-coagulopathy pathways, showed a similar expression pattern to that of CYP20A1*_Alu*-LT. These genes also shared cognate miRNA binding sites for nine miRNAs that were expressed in both conditions and had 10 or more predicted sites on *CYP20A1_Alu*-LT’s 3’UTR. From these findings, we hypothesized that the exonized *Alus* in the 3’UTR of the transcript might have potential miRNA sponging activity. This study opened up many new questions - Is *CYP20A1_Alu*-LT directly mediating the differential expression of 380 genes. Could *CYP20A1_Alu*-LT affect the expression of these genes in other cell types? Does miRNA bind to all the predicted sites? What could be the biological impact of *CYP20A1_Alu*-LT expression in the neurons? However, the high repeat content of the transcript makes functional studies extremely challenging.

In this study, we addressed some of these questions by differentially expressing an Alu-rich fragment of *CYP20A1_Alu*-LT, in the absence of apoptotic or stress triggers. The expression of the transcript was reported to be similar in SK-N-SH and primary neurons in the previous study, hence we used the SK-N-SH cell line here [14]. Surprisingly, we found that the differential expression of *CYP20A1_Alu*-LT has a positive correlation with the expression pattern of the selected genes from the set of 380 genes. We ascertained its miRNA binding and determined a couple of miRNAs that could be mediating this interaction. In conclusion, we report functional evidence for the Alu-rich and miRNA-dense region of 3’UTR of *CYP20A1_Alu*-LT as a miRNA sponge.

## Main text

### Results

#### 3’UTR of the ***CYP20A1_Alu***-LT isoform modulates the expression of selected genes

**Fig. 1:**
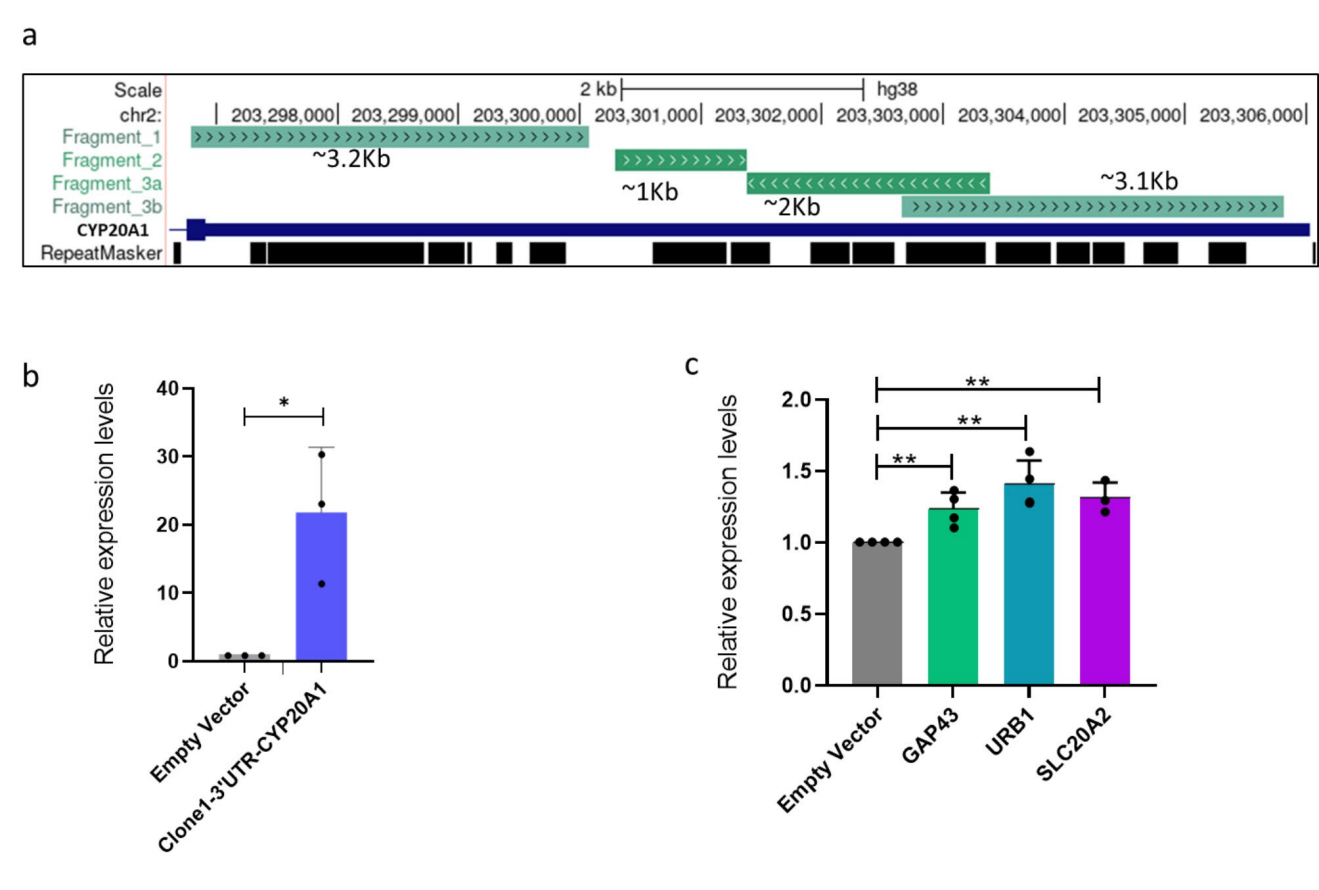
Impact of overexpression of *CYP20A1_Alu-LT* on target genes (a) UCSC representation of different segments of 3’UTR of *CYP20A1_Alu-LT* cloned in the pmiRGlo vector (b) Overexpression of *CYP20A1_Alu-LT* after transfection with Clone1-UTR-CYP20A1 (c) Overexpression of target genes after transfection with Clone1-UTR-CYP20A1

The 3’UTR of *CYP20A1_Alu-*LT is lengthy and has a high density of closely inserted *Alu* repeats, therefore, it was challenging to overexpress the entire transcript. Four segments encompassing the whole UTR region were separately amplified, cloned, and transfected in the SK-N-SH cell line **(**Fig. 1a, Supplementary material). To test the potential sponge function of the 3’UTR of *CYP20A1_Alu*-LT, we cloned fragments of these Alu-rich sequences downstream of the firefly luciferase gene. The introduction of these fragments led to significant downregulation of firefly luciferase signals in all fragments but with a maximum reduction of 91% in clone 1 (Supplementary Figure S1, Supplementary material). Clone 1 (hereafter named Clone1-UTR-CYP20A1), the proximal 1/3rd of the UTR, with 48% of the total miRNA sites (56 out of 116 MREs for the nine miRNAs expressed in primary neurons) and 9 out of the 23 *Alu* repeats was selected for further experiments.

In our previous study, mRNA seq analysis in primary neurons showed upregulation and downregulation in the expression of 380 genes (including *CYP20A1*) after Tat treatment and heat shock, respectively. To ascertain if the overexpression of Clone1-UTR-CYP20A1 in the SK-N-SH cell line could also increase the expression of these genes, a few genes namely, *GAP43, URB1, SLC20A2, NEIL2, EIF4H, MCAM*, and *ORAI2* were selected based on their RNA seq expression levels (Supplementary Table S1). Their expression was checked via qRT-PCR after the transfection of Clone1-UTR-CYP20A1. A ~ 21-fold increase in the expression of the proximal 3’UTR was achieved by Clone1-UTR-CYP20A1 transfection **(**Fig. 1b**)**. Interestingly, a pattern of upregulation in gene expression was observed for all the seven genes that were tested and *GAP43, URB1*, and *SLC20A2* were significantly upregulated **(**Fig. 1c, Supplementary Figure S2a).

**Fig. 2:**
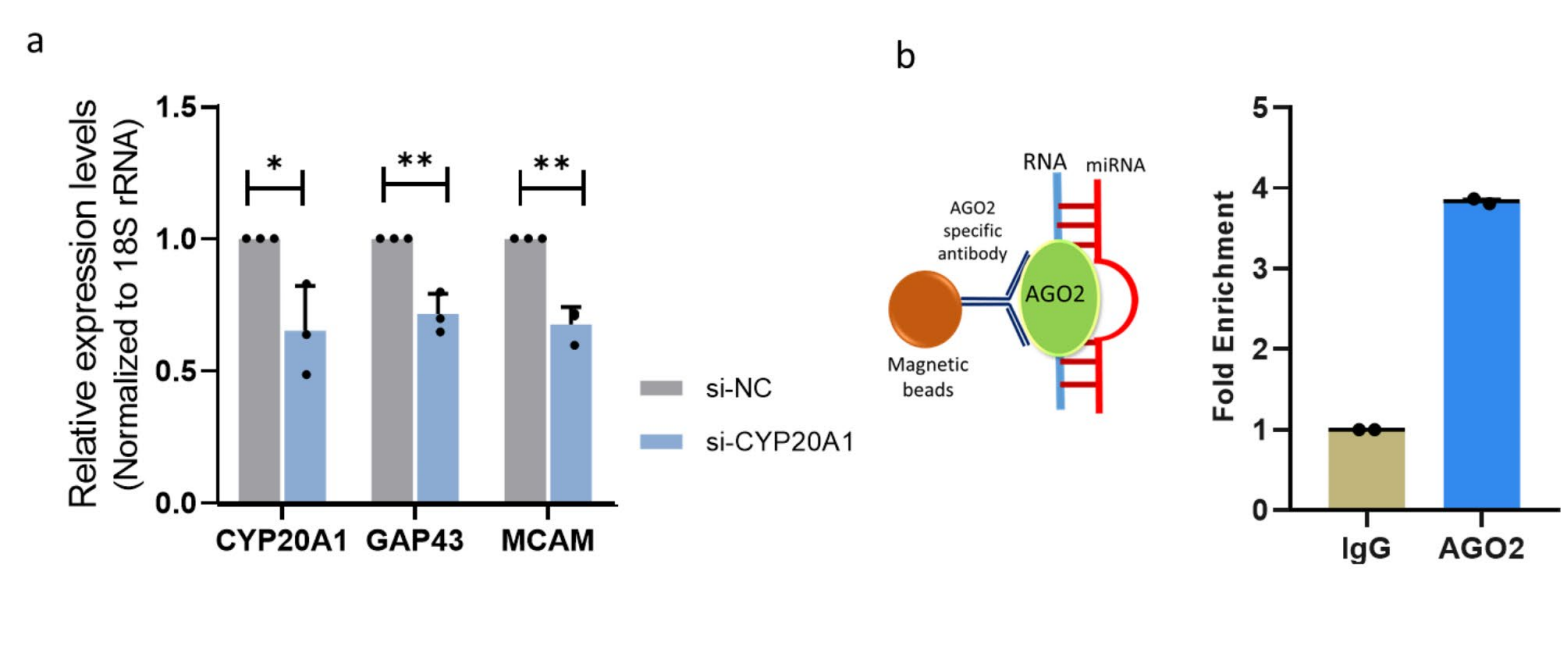
(a) The decreased expression of *CYP20A1_Alu-*LT and target genes after siRNA knockdown (b) Schematic representation of magnetic beads bound with Ago2 antibodies and Ago2-CYP20A1-miRNA complex. The graph represents the enrichment of Clone1-UTR-CYP20A1 in the Ago2 fraction compared to IgG.

In order to strengthen our findings, we performed a knockdown of *CYP20A1_Alu*-LT. siRNAs targeting the 3’UTR of *CYP20A1_Alu*-LT significantly downregulated *CYP20A1_Alu*-LT levels (30%) and also suppressed *GAP43* and *MCAM* expression **(**Fig. 2a**)**. Though the knockdown efficiency was not high it was intriguing to find that all seven genes showed an approximate 20% decrease in the expression after the knockdown of the transcript (Supplementary Figure S2b). This implies that differentially expressed *CYP20A1_Alu*-LT can alter the target gene expression even in the absence of stress triggers.

#### Potential sponge impact could be mediated by binding to miR-619-5p and miR-3677-3p

To ascertain if the Clone1-UTR-CYP20A1 is mediating these effects via binding to miRNAs, we tested its association with RNA-induced silencing complex (RISC). When RISC was enriched using Ago2 immunoprecipitation, there was a 4-fold enrichment of the 3’UTR fragment in Clone1-UTR-CYP20A1 compared to the negative control IgG, confirming the association of the transcript with the miRNA machinery **(**Fig. 2b**).**

**Fig. 3:**
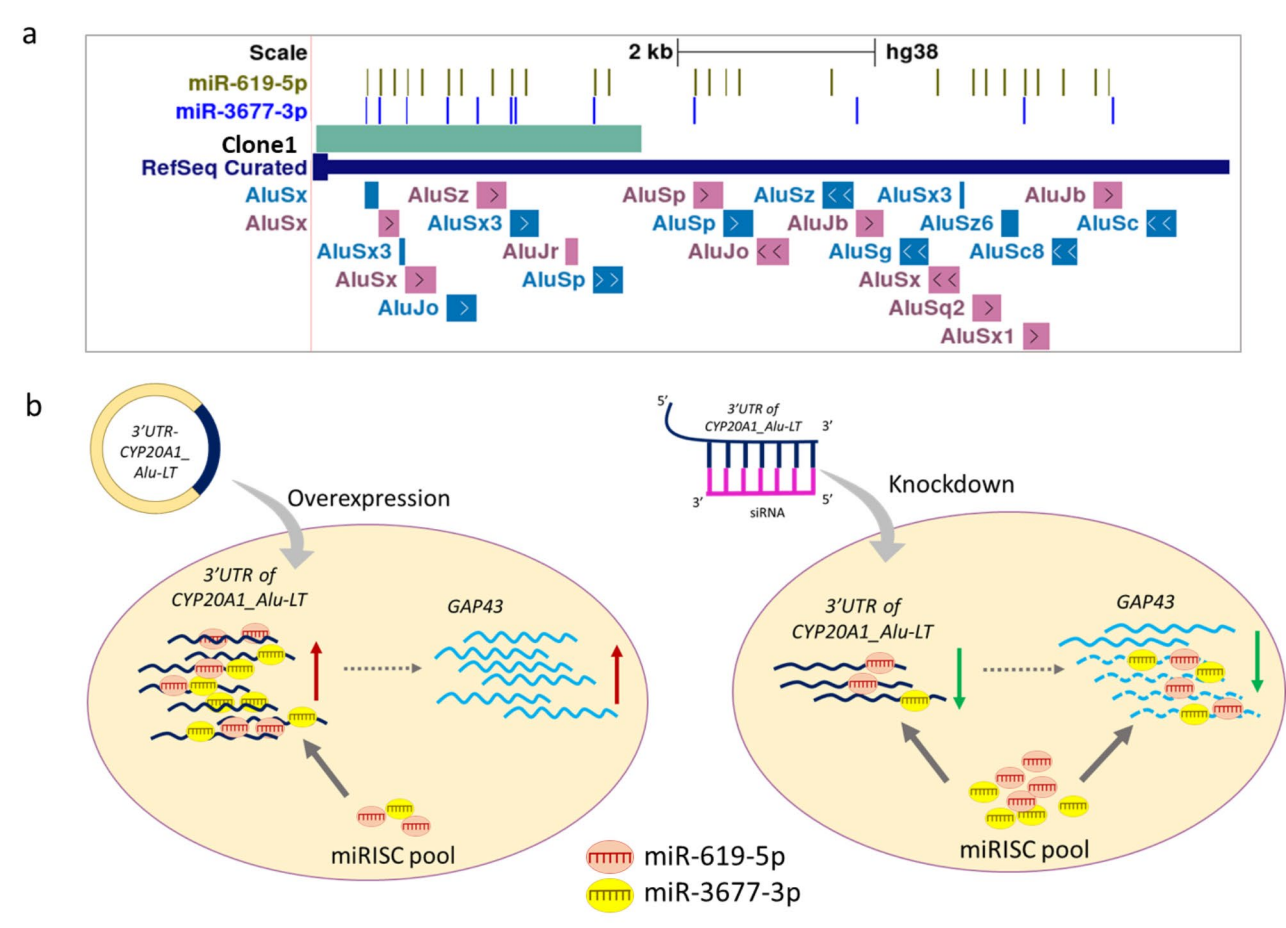
(a) UCSC representation of the 3’UTR of *CYP20A1_Alu_*LT, marked is region of Clone1-UTR-CYP20A1 and miRNA sites. The 23 exonized *Alu* elements present in the 3’UTR are also shown. Arrows represent the orientation of exonized *Alus* (b) Schematic of sponge activity of *Alu* rich 3’UTR of *CYP20A1*_*Alu_*LT and its impact on *GAP43* gene expression. The indirect effect of 3’UTR of *CYP20A1_Alu_*LT on *GAP43* due to its miRNA sponge activity is shown by dotted arrow

Interestingly, *GAP43* (Growth Associated Protein 43) was significantly upregulated as well as downregulated after overexpression and knockdown of *CYP20A1_Alu*-LT, respectively. We earlier predicted miRNA binding sites on *GAP43* using miRanda (version 3.3a). miRanda predicted two potential miRNA binding sites one each for miR-619-5p and miR-3677-3p on the 3’UTR of *GAP43*. However, the 3’UTR of *CYP20A1_Alu*-LT has far more predicted sites i.e. 26 and 12 sites for miR-619-5p and miR-3677-3p, respectively [14]. The region of Clone1-UTR-CYP20A1 alone had 12 and 8 sites for each of the miRNA all on exonized Alus **(**Fig. 3a**)**. Both these miRNAs were expressed in SK-N-SH cell lines at comparable levels (Supplementary Figure S3). Therefore, we hypothesize that *CYP20A1_Alu*-LT might be acting as a molecular sponge for these miRNAs to alter the expression of *GAP43*. When the Alu-rich region is overexpressed, miRNAs would bind more to the *CYP20A1_Alu*-LT than *GAP43*, indirectly leading to increased expression of *GAP43*. However, when the expression of *CYP20A1_Alu*-LT is decreased via siRNA knockdown, the miRNAs are freely available to bind to their target sites on *GAP43* thus degrading the target and decreasing its expression **(**Fig. 3b**)**.

### Discussion

CYP20A1 is a member of the cytochrome p450 protein family that functions as an oxidoreductase enzyme, with moderate activity toward synthetic proluciferin substrates [16]. Several studies in humans and zebrafish have shown that loss or knockdown of *CYP20A1* gene leads to a plethora of behavioral and neurological defects [17–21]. Interestingly, Ensembl shows 9 different transcripts with only one of them having 9 kb long 3’UTR, however, the latest update of NCBI RefSeq reports 18 transcripts each with 9 kb long 3’UTR. Since this gene has 23 *Alu* exonized into its 3’ UTR we were interested in investigating the function of the gene at the transcript level. Previously, we reported that the *CYP20A1_Alu-*LT transcript with 9 kb long 3’UTR has 4742 miRNA binding sites for 994 different miRNAs. In primary neurons, the expression of this transcript increased after apoptotic trigger and decreased after heat-shock treatment. A similar pattern was observed for a set of 380 genes that were enriched in neuro-coagulopathy and shared cognate miRNA binding sites to that of *CYP20A1_Alu-*LT. Therefore, we hypothesized that the *CYP20A1_Alu-*LT might act as a potential miRNA sponge. Nonetheless, the question remained if the correlated gene expression pattern observed was directly mediated by *CYP20A1_Alu-*LT or an effect of different stress triggers.

In this study, we provide functional evidence for the role of *CYP20A1_Alu-*LT as a miRNA sponge in neuronal cells. For a transcript to qualify as a miRNA sponge it has to have certain characteristics. First, it should bind to miRNAs and a higher number of sites are better for its competitive ability. Here, we confirmed that *CYP20A1_Alu-*LT binds to the miRNA machinery, as shown by 4-fold enrichment in Ago2-IP. Second, the expression of the miRNA sponge transcript positively correlates with those of cognate mRNAs. We found that specifically increasing the expression of proximal *Alu* rich and miRNA dense region of *CYP20A1_Alu-*LT significantly increased the expression of *GAP43* and vice-versa was observed upon *CYP20A1_Alu*-LT knockdown. The marginal yet significant change in *GAP43* mRNA expression suggests that *CYP20A1_Alu-*LT might be fine-tuning its expression.

*GAP43* is a neuron-specific protein strongly related to axon growth and regeneration and is reported in many neurodegenerative disorders [22–31]. It is found to have high expression in the brain of humans and other primates and is associated with experience-dependent plasticity [32], [33]. Interestingly, we also reported the expression of *CYP20A1_Alu-*LT in higher primates and even in highly specialized rosehip neurons in humans. Here we propose that the *GAP43* expression might be altered via miR-619-5p and miR-3677-3p. These have > 10 binding sites on *CYP20A1_Alu-*LT which are all present in the exonized *Alus*. It would be interesting to explore if the *CYP20A1_Alu-*LT/GAP43/miRNA axis is playing a role in primate-specific brain functions. Considering *CYP20A1_Alu-*LT has potential binding sites for hundreds of other miRNAs it can act as a sponge in various different conditions, an exciting possibility for future studies. The study provides additional evidence to our previous findings by experimentally showing that exonized Alus in the *CYP20A1* can act as a miRNA sponge. These could act as modifiers and fine-tune the expression of multiple targets by releasing or sequestration of miRNAs. Exonized Alus thus can be one of the important players in the field of miRNA sponges.

### Materials and methods

#### Cloning and overexpression studies

The 3 kb fragment of 3’UTR of *CYP20A1_Alu-LT* (named Clone1-UTR-CYP20A1; Fig. 1a, 3a) was amplified using human genomic DNA and cloned into pmiRGlo Dual-Luciferase miRNA Target Expression Vector (Promega, Cat. No. E1330), downstream of the firefly luciferase gene (FLuc), using XhoI and XbaI enzymes (Supplementary material). The clones were confirmed by sequencing. Lipofectamine-3000 was used to transfect 1.25 µg plasmid/100,000 cells in 12 well plates 24 h post-seeding, followed by a medium change at 6 h and cell lysis 48 h post-transfection. pmiRGlo empty vector was used as control. The cell culture, total RNA isolation, and cDNA synthesis was done as described previously (Bhattacharya et al. 2020, GBE). qRT-PCR data were normalized to the geometric mean of *GAPDH* and 18 S rRNA (N = 3, n = 3).

#### siRNA knockdown

siRNAs targeting 3’UTR of *CYP20A1_Alu-LT* (si-CYP20A1) and a siRNA negative control (si-NC) were designed using InvivoGen siRNA wizard software v3.1 and synthesized by Sigma Aldrich (Supplementary material). ~10^5^ cells were seeded in 12-well plates and 30nM siRNA was transfected 24 h post-seeding. 6 h later the media was changed and cells were harvested 72 h post-transfection. qRT-PCR data were normalized to 18 S rRNA (N = 3, n = 3).

#### RNA immunoprecipitation (RIP) assay

~ 15 million SK-N-SH cells (procured from National Center for Cell Science, Pune, India) were transfected with the Clone1-UTR-CYP20A1. Cells were harvested in cold PBS 48 h post-transfection. RIP was done using the Imprint® RNA Immunoprecipitation Kit (Cat. No. RIP-12RXN, Merck, Sigma) following the manufacturer’s protocols. Antibodies used Ago2 (ab57113, Abcam) and IgG (ab46540, Abcam). RNA isolation was done by adding Trizol directly to the beads. RNA was also isolated from the 10% of Input samples i.e. taken for immunoprecipitation is used for normalization. *CYP20A1* levels in the Ago2, IgG, and Input fractions were checked using qRT-PCR.

### Statistical analysis

GraphPad Prism 8.0.1 (GraphPad Software Inc., CA, USA) was used to plot the graphs and for statistical analyses (Student t-test). Error Bar represents means ± standard deviation (SD). Statistical significance: *p < 0.05, **p < 0.01, ***p < 0.001. ‘N’ refers to biological replicates and ‘n’ refers to technical replicates.

## Limitations

1. The evidences provided in this study are preliminary and more experiments would be required to provide their physiological roles in neuronal function.
2. To confirm the sponge function, direct evidence for miRNA binding to *GAP43* and *CYP20A1_Alu-LT* are required.
3. The physiological impact of miRNA depletion and overexpression in the presence and absence of *CYP20A1_Alu-LT* has not been shown.

## Electronic supplementary material

Below is the link to the electronic supplementary material.

Supplementary Material 1

Supplementary Material 2

## Data Availability

The data generated in the study has been provided in the manuscript and as supplementary files.

## Abbreviations

***CYP20A1***: Cytochrome P450 Family 20 Subfamily A Member 1
***3’UTR***: 3 prime untranslated region
***CYP20A1_Alu-LT***: CYP20A1 *Alu* Long Transcript
***siRNA***: small interfering RNAs
***miRNA***: microRNAs
***GAP43***: Growth Associated Protein 43
***EMT***: epithelial-to-mesenchymal transition
***MREs***: miRNA response elements
***HIV-TAT***: Human immunodeficiency virus - Trans-Activator of Transcription
***URB1***: URB1 Ribosome Biogenesis Homolog;
***SLC20A2***: Solute Carrier Family 20 Member 2
***NEIL2***: Nei Like DNA Glycosylase 2
***EIF4H***: Eukaryotic translation initiation factor 4 H
***MCAM***: Melanoma Cell Adhesion Molecule
***ORAI2***: ORAI Calcium Release-Activated Calcium Modulator 2
***RISC***: RNA induced silencing complex
***IP***: Immunoprecipitation
***AGO2***: Argonaute RISC Catalytic Component 2
***rRNA***: ribosomal RNA
***RIP***: RNA Immunoprecipitation
***IgG***: Immunoglobulin G
