# Supplementary material for: Exonized Alu repeats in the 3’UTR of a CYP20A1_Alu-LT transcript act as a miRNA sponge
Source: BMC Res Notes. 2023 Mar 9;16:32. doi: 10.1186/s13104-023-06289-z (PMC9996890; doi:10.1186/s13104-023-06289-z)
Supplement: Supplementary file 1 — Supplementary Material 1 [file 13104_2023_6289_MOESM1_ESM.docx]

**Supplementary Figure legends**

**Figure S1:** (a) The graph shows the dual luciferase assay results for all the clones of CYP20A1_Alu-LT (N= 3, n=4) (b) The principle of Dual-Luciferase assay is shown to the right. Cloning is done downstream of the firefly luciferase gene such that expression of only firefly luciferase is downregulated due to miRNA binding to the cloned fragment of 3’UTR of CYP20A1_Alu-LT.

**Figure S2** a) Increased expression of the *NEIL2, EIF4H, MCAM*, and *ORAI2* checked via qRT-PCR after overexpression of Clone1-UTR-CYP20A1 b) Decreased expression of the *URB1*, *SLC20A2, NEIL2, EIF4H*, and *ORAI2* checked via qRT-PCR after siRNA mediated knockdown of CYP20A1_Alu-LT.
**Figure S3**: Raw Ct values of miRNA-619-5p and miRNA-3677-3p expression checked in SK-N-SH cell line by qRT-PCR. U1snRNA was used as an internal control.

**Table S1**: RNA seq values of selected genes in primary neurons (pr. Neurons) after Tat treatment and Heat-shock treatment as reported in the previous study (Bhattacharya et al, GBE, 2020).

**Supplementary Material**

**Dual-Luciferase assay:** The firefly luciferase activity (reporter gene for CYP20A1_Alu-LT) and the renilla luciferase activity (internal reference gene for normalization) were determined by the Dual-Luciferase® Reporter Assay System (Promega, E1980) as per the manufacturer’s protocol. Relative luciferase activity = firefly luciferase activity/renilla luciferase activity. Light production was expressed in Relative Light Units (RLU).

Primers used for cloning the 3’UTR of *CYP20A1_Alu-LT.* Marked in red are the restriction enzyme sites XhoI site-CTCGAG in forward primer (FP) and XbaI site-TCTAGA in reverse primer (RP).

| **New _Oligo Name** | **Sequence (5'→3')** | **3’UTR region amplified** | **pmiRGlo Constructs formed** |
| --- | --- | --- | --- |
| CYP_C1_FP | CCGCTCGAGGTATTGGTGAAGAGACTGCA | Fragment 1 | Construct 1 |
| CYP_C1_RP | CTAGTCTAGATTGCTTTAAACTCCACCTCT |  |  |
| CYP_C2_FP | CCGCTCGAGACCAAGTGTCAGATCAGATG | Fragment 2, Fragment 3a | Construct 2, Construct 3a |
| CYP_C2_RP | CTAGTCTAGAGTTGATTGAGCCCTCCTTAA |  |  |
| CYP_C3_FP | CCGCTCGAGTTGTCACTCTGAATGTAGGC | Fragment 3b | Construct 3b |
| CYP_C3_RP | CTAGTCTAGATTCTATGTGCACAGAACACCG |  |  |

Oligo sequences CYP_C2_FP and CYP_C2_RP were used to amplify a single fragment of 3 Kb, but was divided into two pieces that are fragment 2 and fragment 3a due to the presence of an internal restriction enzyme site. However, this did not change our downstream findings or interpretation as only construct 1 (generated from cloning of fragment 1) was used for downstream analysis.

Construct 1 harbored 56 out of 116 miRNA sites (MREs) for the selected nine miRNAs and 9 out 23 *Alu* repeats and yielded a 91% downregulation in DLR assay.

Construct 2 harbored 17 out of 116 miRNA sites (MREs) for the selected nine miRNAs and 3 out of 23 *Alu* repeat and yielded an 85% downregulation in DLR assay.

Construct 3a harbored 13 out of 116 miRNA sites (MREs) for the selected nine miRNAs and 5 out of 23 *Alu* repeats and yielded a 67% downregulation in DLR assay.

Construct 3b harbored 34 out of 116 miRNA sites (MREs) for the selected nine miRNAs and 9 out 23 *Alu* repeats and yielded an 89% downregulation in DLR assay. Construct 3b overlapped proximally with construct 3a and also shared some miRNA sites.

Primers used for qRT-PCR

| **Primer name** | **Sequence(5' to 3')** |  | |
| --- | --- | --- | --- |
| CYP20A1_FP1A | GGCTACACTATTTGTGAAGGATTC | qRT-PCR primers for checking Overexpression |  |
| CYP20A1_RP1 | AAAATAAGCCCACTTGCAGAC |  |  |
| CYP20A1_3'UTR_FP | TTTGTAAGAGCTTCAGGGAA | qRT-PCR primers for checking Knockdown |  |
| CYP20A1_3'UTR_RP | CACCGAACTGTAAACCAATT |  |  |
| GAP43_FP | CAGCCAAGCTGAAGAGAACA |  | |
| GAP43_RP | GGGGATGTGGAAAGCCATTT |  | |
| URB1_FP | AGTGGAGAGAAACGACCTGA |  | |
| URB1_RP | AAAGATCACTTGCTGTCCGC |  | |
| SLC20A2_FP | GTACCAAAGGTGTGCAGTGG |  | |
| SLC20A2_RP | GTACAAACAGCAGGCCAGAC |  | |
| NEIL2_FP | TCAGCAGGTGGTCAAGACAG |  | |
| NEIL2_RP | GGGGCCCCATTTCTTCATCT |  | |
| EIF4H_FP | TTCAGGGCGACATAGATGCT |  | |
| EIF4H_RP | CCATCGTATGTCAAGGCTTCC |  | |
| MCAM_FP | AGACAGGTGTTGAATGCACG |  | |
| MCAM_RP | GTATGAGGACTGGCAGTGGA |  | |
| ORAI2_FP | CGGAAGCTCTACCTGAGCA |  | |
| ORAI2_RP | GGGTACTGGTACTGCGTCTC |  | |
| 18S rRNA_FP | GGCCCTGTAATTGGAATGAGTC |  | |
| 18S rRNA_RP | CCAAGATCCAACTACGAGCTT |  | |
| GAPDH_FP | CGACCACTTTGTCAAGCTCA |  | |
| GAPDH_RP | CTTCCTCTTGTGCTCTTGCTG |  | |
| hsa-miR-619-5p | GCTGGGATTACAGGCATGAGCC |  | |
| hsa-miR-3677-3p | TGGGCTCTGGCCACGGCC |  | |
| hsa-U1snRNA | CGACTGCATAATTTGTGGTAGTGG |  | |
|  |  |  | |
|  |  |  | |
| **siRNA Name** | **Strand** |  | |
| CYP20A1_siRNA | Sense (5'-3') | GGCGGUAGAAGGUUAGAGUAGUU | |
|  | Antisense (3'-5') | UUCUACUCUAACCUUCUACCGCC | |

*Text in red represents overhangs - UU
