# Supplementary material for: Exonized Alu repeats in the 3’UTR of a CYP20A1_Alu-LT transcript act as a miRNA sponge
Source: BMC Res Notes. 2023 Mar 9;16:32. doi: 10.1186/s13104-023-06289-z (PMC9996890; doi:10.1186/s13104-023-06289-z)
Supplement: Supplementary file 2 — Supplementary Material 2 [file 13104_2023_6289_MOESM2_ESM.pptx]

## Slide 1
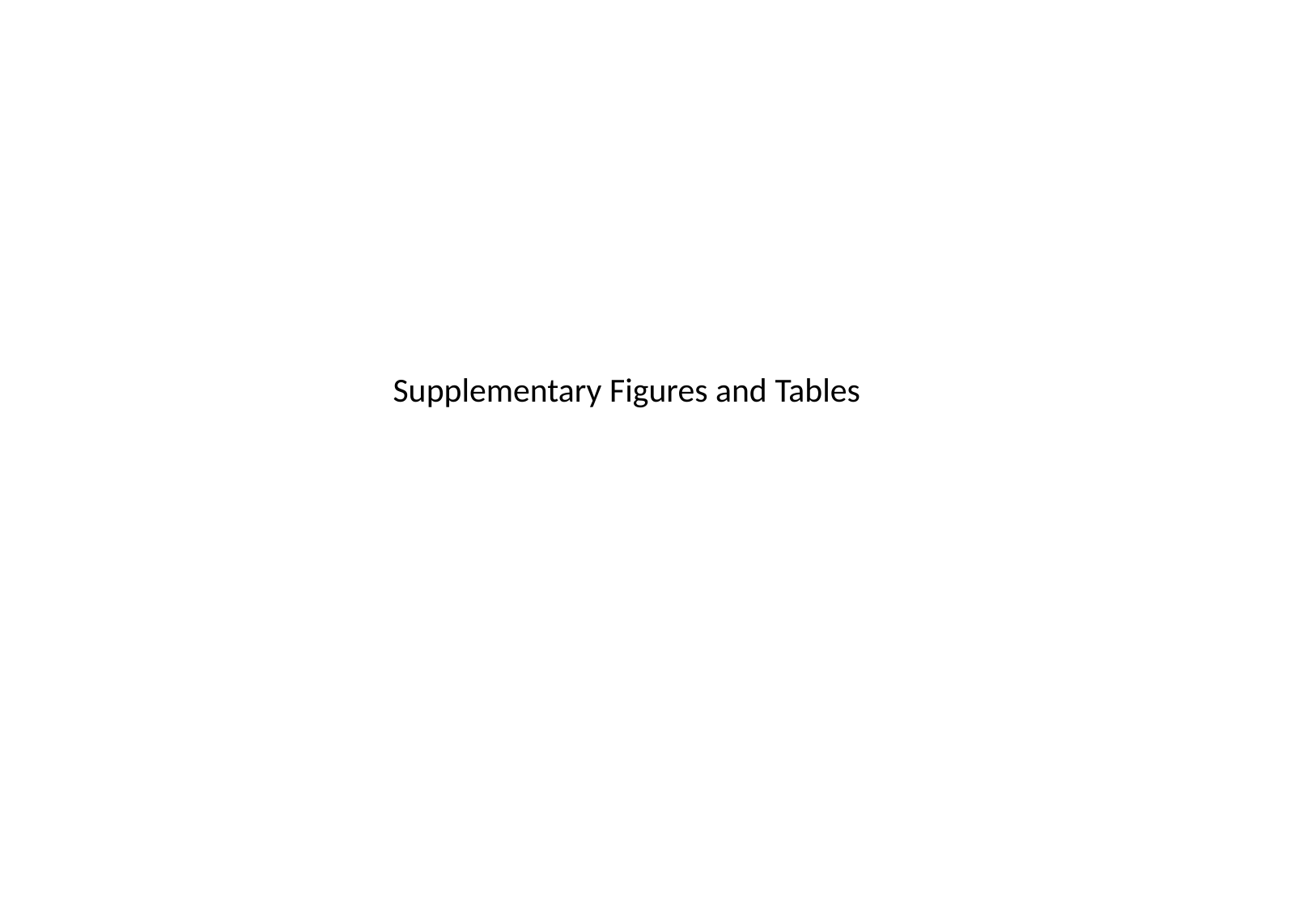

Supplementary Figures and Tables

## Slide 2
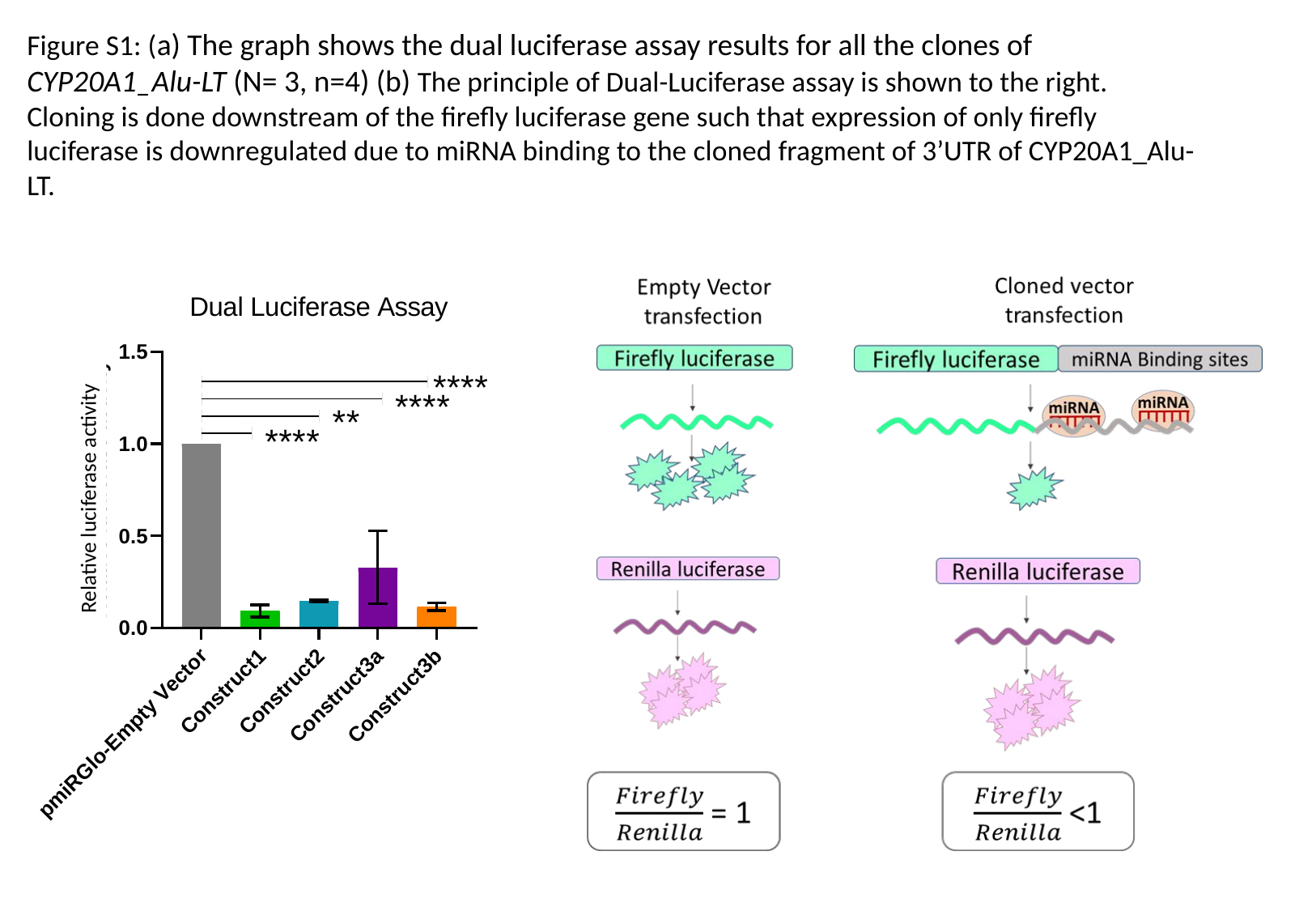

Figure S1: (a) The graph shows the dual luciferase assay results for all the clones of CYP20A1_Alu-LT (N= 3, n=4) (b) The principle of Dual-Luciferase assay is shown to the right.
Cloning is done downstream of the firefly luciferase gene such that expression of only firefly luciferase is downregulated due to miRNA binding to the cloned fragment of 3’UTR of CYP20A1_Alu-LT.
Relative luciferase activity

## Slide 3
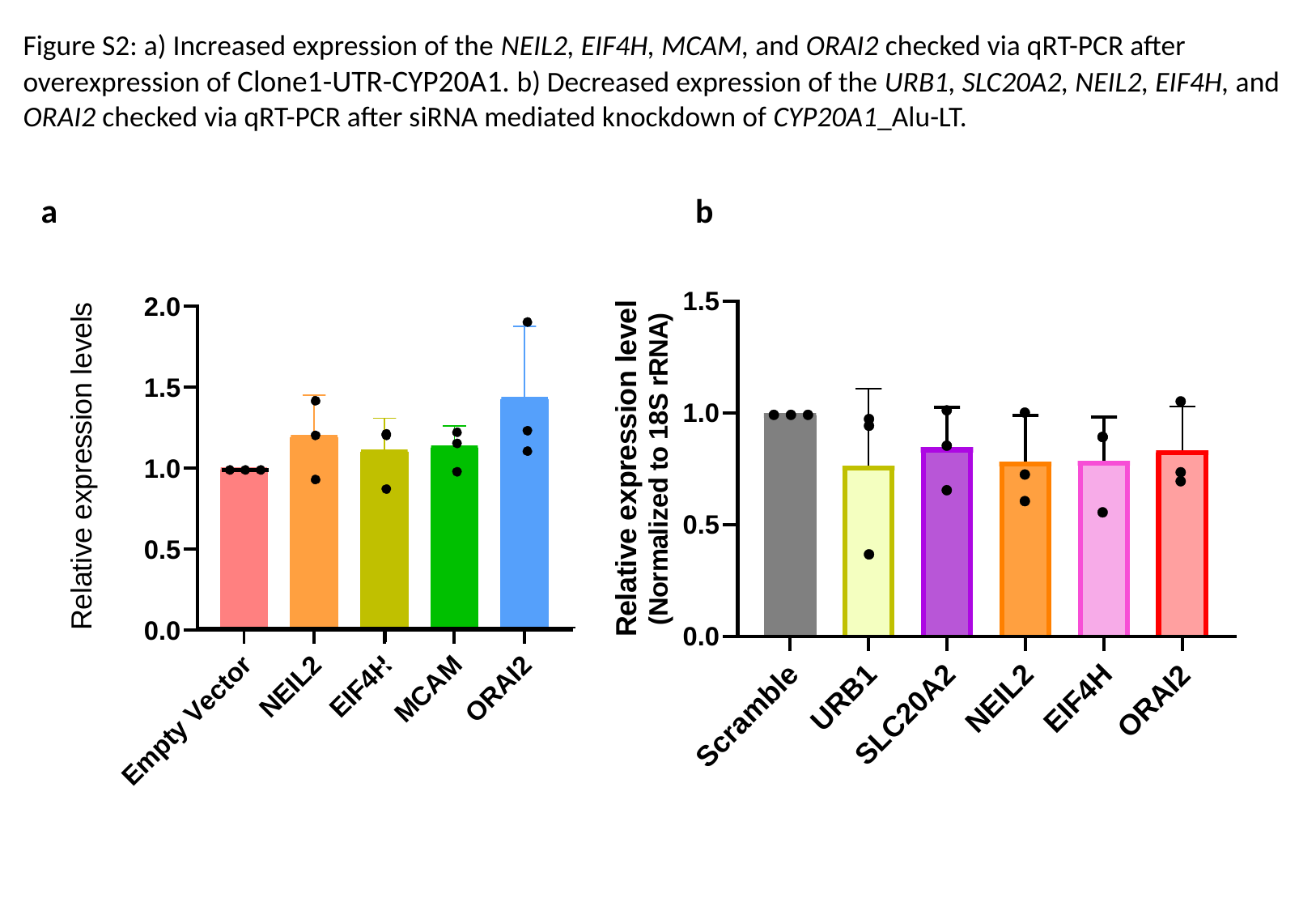

Figure S2: a) Increased expression of the NEIL2, EIF4H, MCAM, and ORAI2 checked via qRT-PCR after overexpression of Clone1-UTR-CYP20A1. b) Decreased expression of the URB1, SLC20A2, NEIL2, EIF4H, and ORAI2 checked via qRT-PCR after siRNA mediated knockdown of CYP20A1_Alu-LT.
a
b

## Slide 4
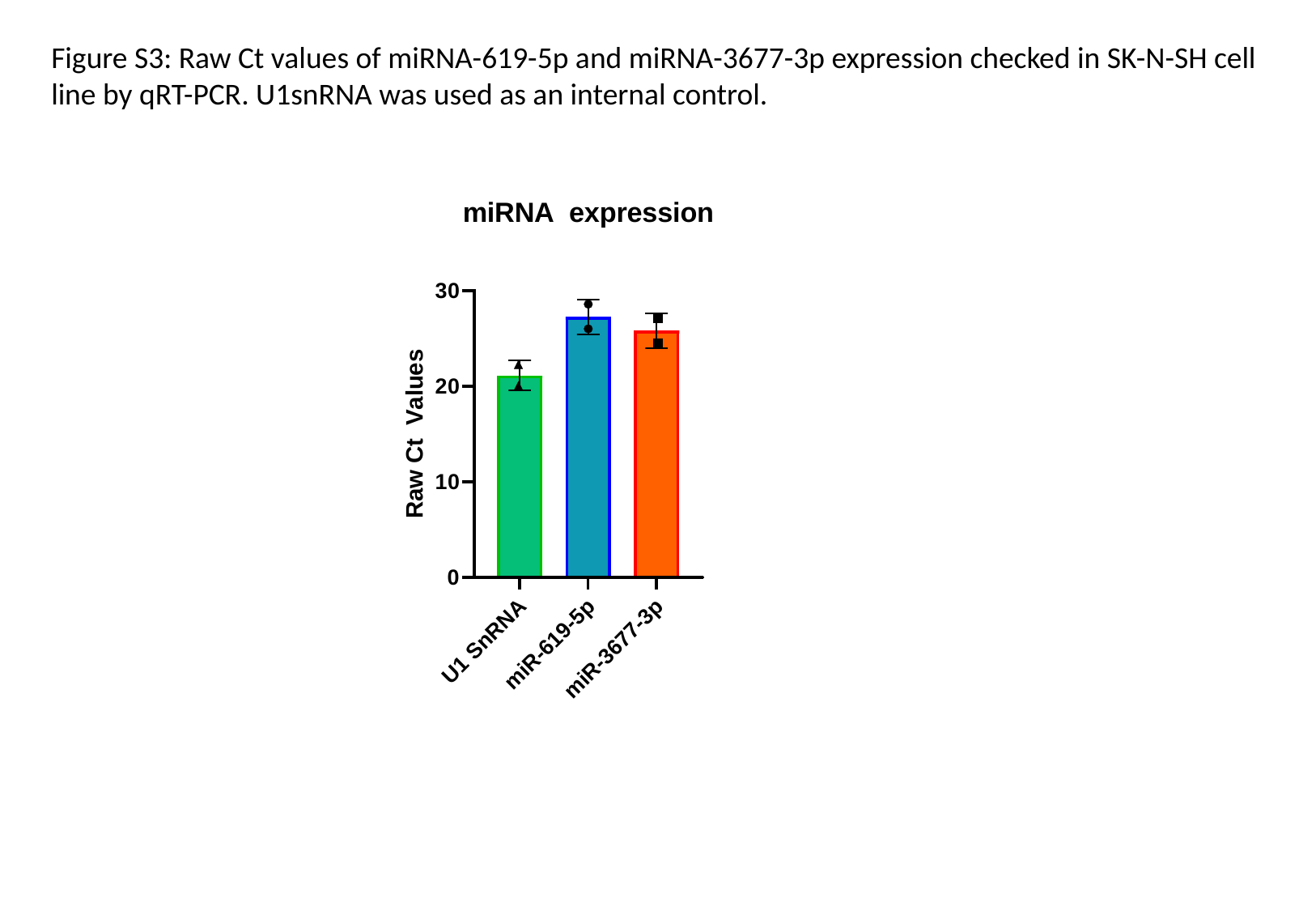

Figure S3: Raw Ct values of miRNA-619-5p and miRNA-3677-3p expression checked in SK-N-SH cell line by qRT-PCR. U1snRNA was used as an internal control.

## Slide 5
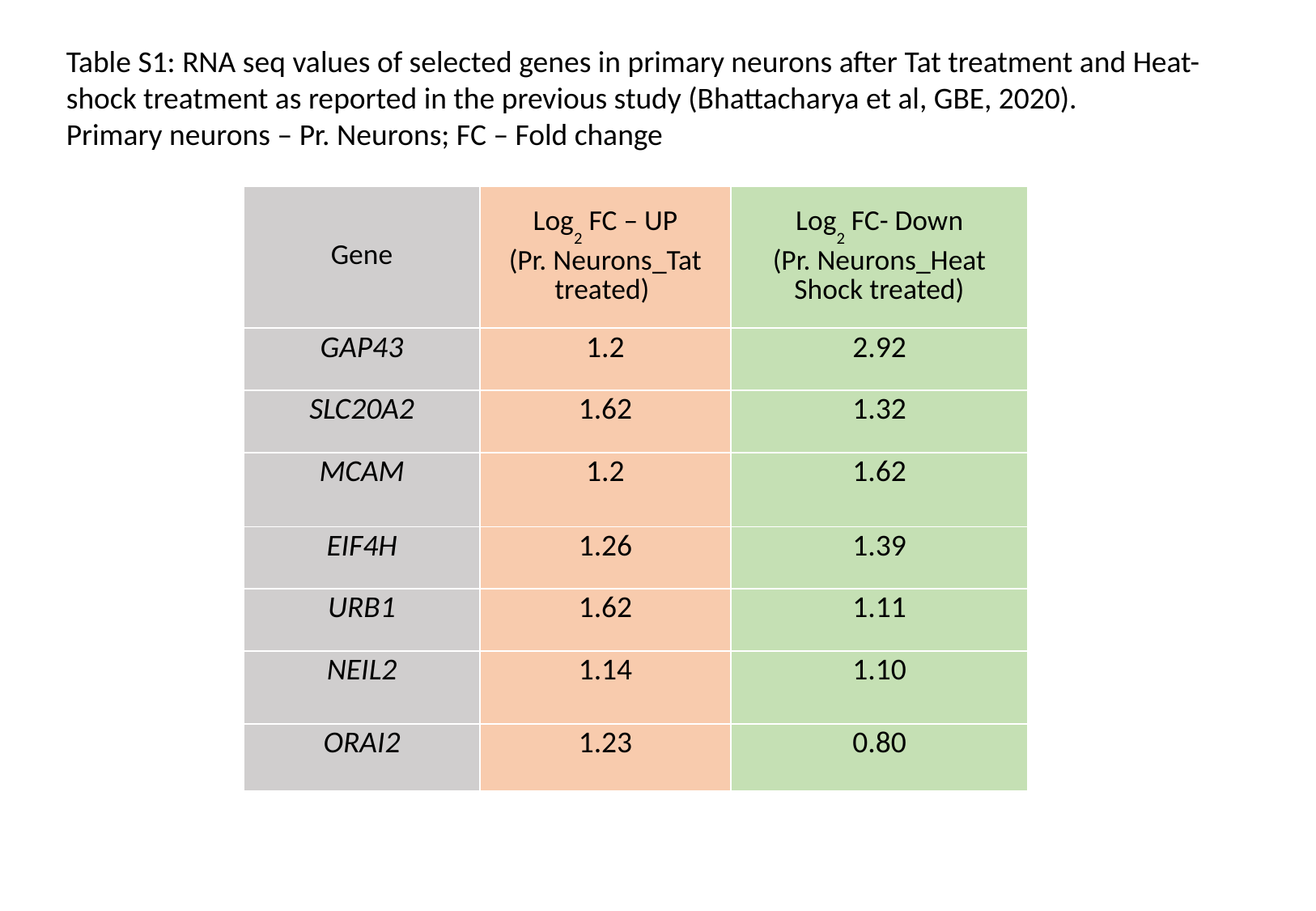

Table S1: RNA seq values of selected genes in primary neurons after Tat treatment and Heat-shock treatment as reported in the previous study (Bhattacharya et al, GBE, 2020).
Primary neurons – Pr. Neurons; FC – Fold change
| Gene | Log2 FC – UP (Pr. Neurons\_Tat treated) | Log2 FC- Down (Pr. Neurons\_Heat Shock treated) |
| --- | --- | --- |
| GAP43 | 1.2 | 2.92 |
| SLC20A2 | 1.62 | 1.32 |
| MCAM | 1.2 | 1.62 |
| EIF4H | 1.26 | 1.39 |
| URB1 | 1.62 | 1.11 |
| NEIL2 | 1.14 | 1.10 |
| ORAI2 | 1.23 | 0.80 |
